# Mushroom Polysaccharides as Potential Candidates for Alleviating Neurodegenerative Diseases

**DOI:** 10.3390/nu14224833

**Published:** 2022-11-15

**Authors:** Xue Jiang, Siqi Li, Xiangru Feng, Lanzhou Li, Jie Hao, Di Wang, Qingshuang Wang

**Affiliations:** 1 College of Life Science and Technology, Changchun University of Science and Technology, Changchun 130022, China; 2 Engineering Research Center of Chinese Ministry of Education for Edible and Medicinal Fungi, Jilin Agricultural University, Changchun 130118, China; 3 School of Life Sciences, Jilin University, Changchun 130012, China

**Keywords:** mushroom polysaccharides, neurodegenerative disease, neuroprotective mechanisms

## Abstract

Neurodegenerative diseases (NDs) are a widespread and serious global public health burden, particularly among the older population. At present, effective therapies do not exist, despite the increasing understanding of the different mechanisms of NDs. In recent years, some drugs, such as galantamine, entacapone, riluzole, and edaravone, have been proposed for the treatment of different NDs; however, they mainly concentrate on symptom management and confer undesirable side effects and adverse reactions. Therefore, there is an urgent need to find novel drugs with fewer disadvantages and higher efficacy for the treatment of NDs. Mushroom polysaccharides are macromolecular complexes with multi-targeting bioactivities, low toxicity, and high safety. Some have been demonstrated to exhibit neuroprotective effects via their antioxidant, anti-amyloidogenic, anti-neuroinflammatory, anticholinesterase, anti-apoptotic, and anti-neurotoxicity activities, which have potential in the treatment of NDs. This review focuses on the different processes involved in ND development and progression, highlighting the neuroprotective activities and potential role of mushroom polysaccharides and summarizing the limitations and future perspectives of mushroom polysaccharides in the prevention and treatment of NDs.

## 1. Introduction

Human life expectancy has increased rapidly owing to the improvements in diet, sanitation, and medicine. The decline in immunocompetence and the generation of chronic inflammation associated with age is a causative factor in the development of neurodegenerative diseases (NDs) [1]. Individuals with NDs, such as Parkinson’s disease (PD), Alzheimer’s disease (AD), amyotrophic lateral sclerosis (ALS), Huntington’s disease (HD), Lewy body dementia (LBD), and frontotemporal dementia (FTD), display diverse pathophysiological symptoms. Some NDs may cause memory and cognitive impairments, while others may weaken a person’s ability to move, breathe, and speak [2]. NDs are characterized by large inter-individual differences, complex etiologies, and multiple clinical manifestations. However, the basic feature of NDs is the progressive loss of specific neurons [3]. A variety of mechanisms, including neuroinflammation, oxidative stress (OS), autophagic dysfunction, apoptosis, and excitatory amino acid toxicity, have been reported to participate in the pathological process of NDs [4]. Thus, protecting neurons is the key to the treatment of NDs.

AD is a complex neuropathological disease characterized by intracellular neurofibrillary tangles and extracellular amyloid plaques [5]. Patients with AD exhibit impaired cognitive judgment and memory which is usually accompanied by disorientation, mood swings, and delirium [6]. At present, the main drugs for the treatment of AD include cholinesterase inhibitors (such as galantamine, memantine, rivastigmine, donepezil, and tacrine) [7], antioxidant drugs (such as melatonin and monoamine oxidase inhibitors) [8,9], calcium channel blockers (such as flunarizine, nimodipine, nilvadipine, and nitrendipine) [10], and drugs directed at β1 amyloid (such as MK-8931 and E2069) [11]. PD, another severe ND, conferring symptoms such as rigidity, bradykinesia, tremor, and postural instability, is characterized by the accumulation of misfolded α-synuclein and by the loss of dopaminergic neurons in the substantia nigra pars compacta [12,13]. PD remains an incurable disease, despite significant progress made over the last couple of decades. Current therapies for PD mainly include catechol-O-methyltransferase inhibitors (such as entacapone, tolcapone, and opicapone) [14], drugs for stimulating dopaminergic signaling (e.g., DOPA decarboxylase inhibitors and levodopa) [15], monoamine oxidase B inhibitors (such as rasagiline and selegiline) [16] and dopamine agonists [17]. HD is an autosomal dominant condition characterized by degeneration of the striatum and general shrinkage of the brain; symptoms include motor impairment (such as loss of coordination and chorea) and psychiatric conditions (such as obsessive compulsive disorder, psychosis, and depression) [18]. Currently available drugs for HD include inhibitors of vesicular monoamine transporter type 2 (such as deutetrabenazine and tetrabenazine), which can improve chorea by depleting dopamine in presynaptic terminals [19]. ALS is a type of motor neuron disease characterized by selective damage to the lower and upper motor neurons [20]. Riluzole and edaravone are two drugs currently approved for the treatment of ALS [21]. The side effects of these drugs, in the treatment of NDs, are summarized in Table S1. Current drugs for the treatment of NDs mainly concentrate on symptom management, and undesirable side effects and adverse reactions are common. As no cure is available for these devastating diseases [6], it is necessary to find alternative natural agents with fewer side effects for the treatment of NDs.

Mushroom polysaccharides exhibit various pharmacological activities, including multi-targeting bioactivities, have low toxicity and high safety, and are relatively cheap. For these reasons, they have attracted the attention of many researchers [4]. Polysaccharides extracted from mushrooms have enormous potential to inhibit the progression of various NDs, and studying the therapeutic effects of mushroom polysaccharides on NDs has become a crucial area of research [22]. *Ganoderma lucidum* polysaccharides exhibit significant neuroprotective effects in cerebellar granule cells by regulating the expression of proteins associated with apoptosis and inhibiting neuronal apoptosis induced by OS [23]. Polysaccharides isolated from *Antrodia camphorata* can increase the activities of antioxidant enzymes and inhibit the expression of reactive oxygen species (ROS)-nucleotide-binding domain, leucine-rich-containing family, and pyrin domain containing 3 (NLRP3) in the substantia nigra-striatum, thereby protecting dopaminergic neurons and improving the exercise capacity of PD mice induced by 6-hydroxydopamine (6-OHDA) [24]. In an amyloid precursor protein/presenilin 1 (APP/PS1) mouse model, *Grifola frondosa* polysaccharides have been shown to activate astrocytes and microglia, promote the recruitment of microglia to amyloid beta (Aβ) plaques, enhance Aβ phagocytosis, and eventually alleviate memory and learning impairment through immunomodulation [25]. *Dictyophora indusiata* polysaccharides have displayed neuroprotective activities in transgenic *Caenorhabditis elegans* HD models through the reduction of ROS levels and the alleviation of chemosensory behavior deficits [26]. Numerous mushroom polysaccharides have been shown to exhibit neuroprotective effects in different models of neurodegeneration, in vivo and in vitro. Therefore, finding and studying effective mushroom polysaccharide substances for the treatment of NDs may present a promising alternative to conventional therapeutics.

In this review, we summarize the mechanisms of NDs and the neuroprotective effects of mushroom polysaccharides and discuss the potential of mushroom polysaccharides as new therapeutic strategies for the treatment of NDs. We hope our summary provides a basis for the use of mushroom polysaccharides as nutraceutical drugs against NDs.

## 2. Retrieval Strategy

This is an extensive review of the literature on the beneficial roles of mushroom polysaccharides in different NDs. We searched NCBI databases and the Web of Science to retrieve studies using the following keywords: mushroom polysaccharide, neurodegenerative diseases, neuroprotective, oxidative stress, amyloid formation, neuroinflammation, cholinesterase, mitochondrial dysfunction, apoptosis, neurotoxins and ferroptosis. Articles published from 2008 to 2022 were included and selected by reading titles and abstracts. Reference lists of articles were also reviewed for additional relevant studies. Related studies conducted on any species were included, and non-scientific experiments or review articles were excluded.

## 3. Mechanisms of NDs

**Figure 1:**
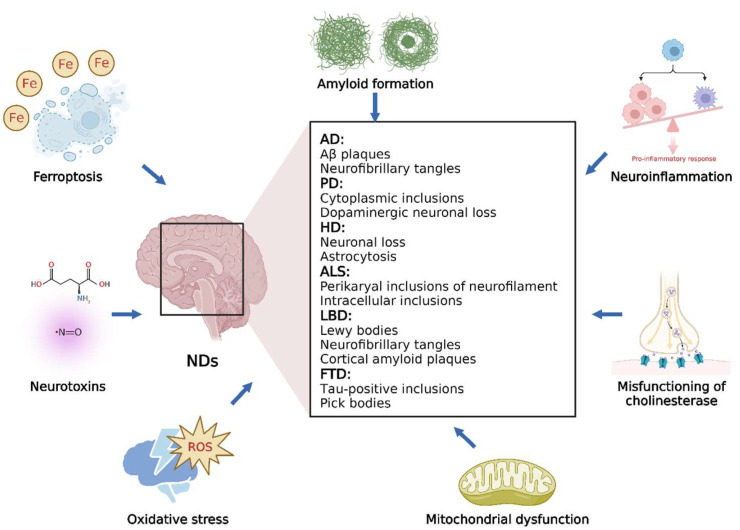
Overview of the main NDs and related processes.

Neurodegeneration is a complex process that can lead to neuronal dysfunction. Various extracellular and intracellular mechanisms involved in different biochemical pathways result in neurodegeneration in a synergistic or additive manner, which must be considered when developing effective therapies for NDs. In this section, we briefly discuss several common NDs and different processes that significantly affect neurodegeneration (Figure 1).

### 3.1. Overview of NDs

NDs are defined as debilitative and fatal conditions leading to exacerbated death of nerve cells; these mainly include AD, PD, HD, ALS, LBD, and FTD [27]. AD, the most common among all NDs, is characterized by the aggregation of neurofibrillary tangles and accumulation of Aβ deposits in the brain [28]. PD is histopathologically characterized by cytoplasmic inclusions composed of insoluble protein aggregates and a serious loss of dopaminergic neurons, which results in progressive movement disorders, such as rigidity, bradykinesia, and tremor. HD is an autosomal dominant condition caused by neuronal degeneration in cortical regions, resulting in psychiatric symptoms, involuntary movements, and dementia [18,27]. ALS is characterized by rapid progressive degeneration of motor neurons in the spinal cord and brain. Intracellular inclusions (such as Lewy body-like cytoplasmic inclusions and Bunina bodies) and perikaryal inclusions of neurofilaments are the neuropathological features of ALS [20,27]. In older individuals, LBD is a common type of degenerative dementia and is clinically characterized by recurrent visual hallucinations, progressive cognitive impairment, and Parkinsonism [27]. The presence of Lewy bodies in the cortex and subcortex is the main histological characteristic of LBD, and some LBD patients also exhibit the pathology of AD, such as neurofibrillary tangles and cortical amyloid plaques [29]. As a heterogeneous group of syndromes, FTD results from neurodegeneration in the temporal or frontal lobes. Affected neurons usually exhibit tau-positive inclusions, which differ from neurofibrillary tangles in AD [30].

### 3.2. Different Processes Involved in NDs

#### 3.2.1. OS

OS is caused by the overproduction of ROS and the inability of biological systems to repair the resulting damage and detoxify these species. OS has been demonstrated to play a ubiquitous role in the pathogenesis of NDs [31]. Neurons are particularly vulnerable to oxidative damage due to their inefficient antioxidant systems, high polyunsaturated fatty acid content in membranes, and high oxygen consumption [32]. As a by-product of metabolism, ROS participates in the degeneration of nerve cells through the regulation of various biological molecules (such as proteins, lipids, DNA, and RNA), and the uncontrolled and excessive production of ROS induced by OS can destroy cellular structures and biomolecules, finally resulting in neuronal death [33]. In addition, the central nervous system (CNS) is highly sensitive to OS due to its high consumption of oxygen and the presence of abundant metals and fatty acids [34]. In general, OS results in lipid oxidation in the brain and plays an important role in the progression of NDs [35].

#### 3.2.2. Amyloid Formation

Protein folding is the process by which proteins form three-dimensional structures and have biological functions that are crucial for human health [36]. With the help of chaperone proteins, misfolded proteins are refolded or degraded; however, continuous protein misfolding without proper clearance can lead to the formation of deposits and oligomers that further result in NDs, such as PD, HD, AD, ALS, and transmissible spongiform encephalopathies [35,36]. Many studies have revealed a common pathogenic mechanism related to these NDs: misfolded proteins accumulate in different areas of the brain, leading to amyloidosis of the CNS [37,38].

#### 3.2.3. Neuroinflammation

Neuroinflammation, a complex reaction of the CNS to a variety of factors (such as trauma, degeneration, infection, and toxins), is related to brain homeostasis [4]. However, uncontrolled or protracted neuroinflammation can lead to neuronal death [39]. As innate immune cells in the CNS, microglia are activated in several different states under pathological and physiological conditions and play an important role in neuroinflammatory responses [40]. Excessive activation of microglia releases various pro-inflammatory cytokines, resulting in synaptic dysfunction, neurogenesis inhibition, and neuronal death [41].

#### 3.2.4. Misfunctioning of Cholinesterase

Acetylcholine (ACh) is an important chemical signaling molecule that controls the concentration of transmitters at the synapse [35]. Choline acetyltransferase (ChAT) and acetylcholinesterase (AChE) are the synthetic and hydrolytic enzymes of ACh [42]. AChE belongs to the cholinesterase family, which can metabolize choline esters and is critical for proper functioning of the nervous system. AChE breaks synaptic transmission through the hydrolysis and inactivation of ACh released by cholinergic nerve endings [35]. In specific areas of the nervous system, changed levels of Ach or altered expression and function of cholinergic receptors are closely correlated with multiple NDs, such as PD, HD, and AD [43].

#### 3.2.5. Mitochondrial Dysfunction

Mitochondrial dysfunction has been shown to occur in the most disabling and prevalent NDs, such as PD, AD, HD, ALS, and spinal muscular atrophy [44]. Intracellular ROS production mainly occurs in the mitochondria during electron transport [45]. The broken mitochondria cause excessive production and release of ROS as well as a decrease in oxidative metabolism enzymes, such as cytochrome oxidase and pyruvate dehydrogenase complex [35]. Functional and morphological changes in mitochondria influence various processes, including the alteration of calcium homeostasis, reduction of adenosine triphosphate (ATP), and induction of apoptosis, which lead to decreased brain energy [44].

#### 3.2.6. Neurotoxins

As a category of exogenous chemicals, neurotoxins have a negative influence on the function of the premature and mature nervous systems and have destructive effects on nervous tissue because of the explicit target of neural components [35]. Common neurotoxins include nitric oxide (NO), tetrodotoxin, botulinum toxin, tetanus toxin, ethanol, lead, and manganese glutamate [46]. Certain chemical structures (such as NO and glutamate) are crucial for normal body function; however, they can become neurotoxic at high concentrations. For example, 6-OHDA and 1-methyl-4-phenyl-1,2,3,6-tetrahydropyridine induce the development of NDs through inflammation and OS [47,48].

#### 3.2.7. Ferroptosis

Ferroptosis is a form of iron-dependent regulated cell death and occurs through the lethal accumulation of lipid-based ROS when glutathione (GSH)-dependent lipid peroxide repair systems are compromised, which can be inhibited and reversed by lipophilic antioxidants and iron chelators [49,50]. The increase in free intracellular iron, oxidation of membrane polyunsaturated fatty acids, and depletion of the redox glutathione peroxidase 4 (GPx4)/GSH ratio are the three main factors related to cell death in ferroptosis [51,52]. Iron overload can lead to lipid peroxidation in astrocytes, microglia, neurons, Schwann cells, and oligodendrocytes. Moreover, low activities of the glutathione system and GPx4 were confirmed to be related to ferroptosis in motor neurodegeneration [51]. Ferroptosis is consistent with some established and salient features of NDs, such as iron dyshomeostasis and lipid peroxidation, indicating that ferroptosis may be involved in the progression of NDs.

## 4. Neuroprotective and Therapeutic Effects of Mushroom Polysaccharides

**Figure 2:**
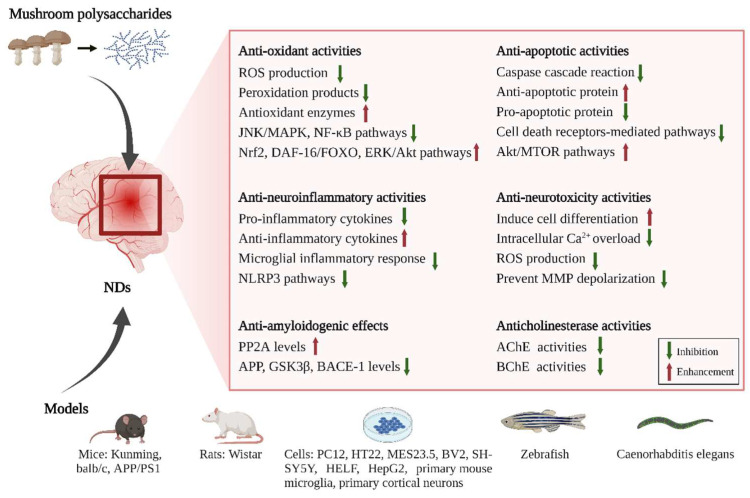
The neuroprotective mechanisms of mushroom polysaccharides involved in NDs.

In this section, we focus on the preventive and/or protective effects of mushroom polysaccharides on NDs (Table 1 and Figure 2). According to recent studies, excessive ROS production, amyloid formation, cholinesterase dysfunction, mitochondrial dysfunction, and ferroptosis are involved in neurodegeneration. All these events separately and/or together result in uncontrolled inflammation and oxidation of neurons, which finally cause neuronal deterioration in the nervous system. In recent years, mushroom polysaccharides have been reported to exhibit neuroprotective effects and ameliorate memory, learning, cognitive, and motor impairment by regulating the above processes in various models, both in vivo and in vitro. They, therefore, may have potential as therapeutic agents for NDs.

### 4.1. Antioxidant Activities of Mushroom Polysaccharides

OS and chronic inflammation are intertwined pathological processes in NDs [53]. The overproduction of ROS can regulate inflammatory pathways and induce the expression of pro-inflammatory factors, which exhibit stimulatory effects on brain inflammation [48]. In turn, increased inflammation can further promote ROS production through various pathways such as nuclear factor kappa-B (NF-κB) signaling [53]. Proteins modified by ROS are conducive to the formation of aggregates; hence, OS also accelerates the aggregation of pathogenic proteins [54]. Owing to the crucial role of OS in NDs, antioxidant polysaccharides from various mushrooms have been shown to improve cognitive and motor functions and attenuate neuronal damage in various neurodegenerative models.

Mushroom polysaccharides can reduce ROS production and related peroxidation product levels and increase various antioxidant enzyme activities in multiple models of NDs. TLH-3, a polysaccharide from *Tricholoma lobayense* with a molecular weight of 4.23 kDa, conferred anti-aging effects by decreasing the levels of ROS and the breakdown product of lipid peroxidation malondialdehyde (MDA) in d-galactose (Gal)-treated Kunming mice [55]. A study reported the neuroprotective effects of *Ganoderma atrum* polysaccharide (PSG-1) and its possible mechanisms in a D-Gal-induced aging mouse model. PSG-1 consists of glucose (Glc), Gal, and mannose (Man) in a molar ratio of 4.91:1.28:1 with a molecular weight of 1013 kDa and was found to ameliorate aging-associated pathologies by increasing the activities of antioxidant enzymes, including catalase (CAT), superoxide dismutase (SOD), GSH, and GPx [56]. In addition, polysaccharides obtained from *Dictyophora indusiate*, *Pleurotus ostreatus*, *Auricularia auricula-judae*, and *Flammulina velutipes* were also confirmed to exhibit neuroprotective effects through the reduction of ROS and peroxidation product levels and the enhancement of antioxidant enzyme activities [26,57,58,59].

Furthermore, mushroom polysaccharides exert neuroprotective effects via the regulation of OS-related signaling pathways. ACPS, a polysaccharide isolated from *Amanita caesarea*, was confirmed to exhibit significant anti-AD effects and neuroprotective activities via the regulation of nuclear factor erythroid 2-related factor 2 (Nrf2)-mediated OS in both L-glutamine (Glu)-exposed HT22 cells and in AD mice induced by D-Gal and AlCl_3_ [60]. IOPS, another polysaccharide purified from *Inonotus obliquus* with a molecular weight of 111.9 kDa, also demonstrated antioxidative effects through regulation of Nrf2 signaling in both L-Glu-damaged HT22 cells and APP/PS1 transgenic mice [61]. Decay-accelerating factor-16 (DAF-16)/forkhead box O (FOXO) is a crucial mediator of OS, and regulating the DAF-16/FOXO pathway is a feasible way to reduce oxidative damage [62]. A type of *D. indusiata* polysaccharide, mainly consisting of Man, fucose (Fuc), Glc, Gal, rhamnose (Rha), glucuronic acid (Glc-UA), and xylose (Xyl), showed antioxidant and neuroprotective activities by regulating the DAF-16/FOXO pathway in C. elegans models [26]. In addition, *G. lucidum* polysaccharides have been shown to regulate NDs, such as AD and epilepsy, by modulating OS/related pathways, including extracellular regulated protein kinase (ERK)/protein kinase B (Akt) signaling, c-Jun N-terminal kinase (JNK)/mitogen activated protein kinase (MAPK) signaling, and NF-κB signaling [63,64,65].

### 4.2. Anti-Amyloidogenic Effects of Mushroom Polysaccharides

Different amyloid fibrils produced by internally disordered, inappropriately folded proteins are closely related to some NDs. Irregular folding and aggregation of Aβ is one of the main neuropathological features of AD [35]. Mushroom polysaccharides targeting Aβ proteins may be promising therapeutic agents. Both decreased Aβ degradation and increased Aβ production result in AD [66]. Amyloid precursor protein (APP), a transmembrane protein in the brain, protects the nervous system by regulating intracellular calcium homeostasis and synaptic transmission [67]. Transmembrane APP can be cleaved by β- and γ-secretases to produce Aβ [68]. β-secretase is the rate-limiting enzyme in the process of Aβ production, and its primary component is beta-secretase 1 (BACE-1); hence, BACE-1 activity is crucial to the production of Aβ [69].

Polysaccharides isolated from *P. ostreatus* ameliorated cognitive impairment induced by D-Gal and Al in an AD rat model by decreasing Aβ formation and tau phosphorylation via elevation of PP2A expression and reduction of APP, glycogen synthase kinase 3beta (GSK3β), and BACE-1 expression [57].

### 4.3. Anti-Neuroinflammatory Activities of Mushroom Polysaccharides

Neuroinflammatory disorders (NDs), such as AD and PD, can be initiated and enhanced by neuroinflammation. Activation of astrocytes and microglia defends against damaged tissues and harmful pathogens, while their prolonged activation leads to neuroinflammation, thereby triggering and promoting neurodegeneration [70]. Currently, no therapies have been developed to stop the progression of neurodegeneration. Mushroom polysaccharides have been reported to exhibit potential anti-neuroinflammatory activities through the inhibition of over-activated microglia, reduction of released pro-inflammatory factors, and regulation of relevant pathways.

*G. lucidum* polysaccharides (GLP) promote the expression of anti-inflammatory cytokines and downregulate the expression of pro-inflammatory cytokines in BV-2 and primary microglia induced by lipopolysaccharide or Aβ. In addition, GLP modulated microglial morphology, migration, and phagocytosis, which are related to inflammation, in the brain of zebrafish, indicating that the neuroprotective effect of GLP was achieved through regulation of the microglial inflammatory response [71]. The anti-neuroinflammatory effects *of A. camphorata* polysaccharides (APC) were studied in 6-OHDA induced PD mice models, and the results showed that the motor symptoms of PD mice were improved by reducing the expression of NLRP3 and its related downstream inflammatory factors after APC treatment [72]. In another study, APC was found to exert neuroprotective effects via inhibition of inflammation-related ROS-NLRP3 pathways in both cell and mouse models of PD induced by 6-OHDA [24]. Although the structural characteristics and composition of ACP are unclear, these findings indicate the potential of APC in PD therapy.

### 4.4. Anticholinesterase Activities of Mushroom Polysaccharides

AChE inhibitors can increase ACh levels at the synapse, thereby enhancing cholinergic activity in the brain [73]. Currently, four AChE inhibitors (memantine, rivastigmine, galantamine, and donepezil) are used to ameliorate cognitive deficits in NDs and prevent dementia. However, these agents have undesirable side effects such as bradycardia, liver toxicity, dizziness, and bowel disturbances [35]. Hence, there is a great need for effective anticholinesterase polysaccharides obtained from mushrooms for the improvement of NDs.

Polysaccharides isolated from two mushrooms (*Coprinellus truncorum* and *Coprinus comatus*) mainly contain β-glucans, which were screened in liquid and found to display AChE inhibitory activities, revealing the possible use of the screened mushrooms in AD treatment [74]. Another study confirmed the AChE and butyrylcholinesterase (BChE) inhibitory activities of *Fuscoporia torulosa* polysaccharides using spectrophotometry [75]. In addition, mushroom polysaccharides isolated from *Morchella esculenta*, which are considered promising therapeutic agents for PD and AD treatment, showed substantial inhibitory effects on AChE and BChE [76,77]. The inhibitory activities of polysaccharides from French and Iranian strains of *Agaricus subrufescens* were investigated for AChE and BChE in another study involving AD, and the results suggested that both polysaccharides showed selective AChE inhibitory effects, and their anti-Aβ aggregation activities were comparable to those of the reference drug, donepezil [77]. Therefore, mushroom polysaccharides are expected to be developed as a new class of AChE inhibitory agents with therapeutic action against NDs such as PD and AD.

### 4.5. Anti-Apoptotic Activities of Mushroom Polysaccharides

Apoptosis plays a crucial role in the development of the nervous system. The resistance of neurons to apoptosis in the CNS of adults prevents a massive loss of neurons under normal physiological conditions [78]. In NDs, apoptosis is the main pathway of neuronal death and the expression of critical proteins relevant to apoptosis is significantly altered [79]. According to recent studies, the main mechanisms by which mushroom polysaccharides inhibit nerve cell apoptosis are as follows: (1) they block apoptotic pathways mediated by mitochondria by preventing the cascade reaction of caspase and maintaining the stability of the mitochondrial internal environment. In addition, they promote anti-apoptotic protein expression and inhibit pro-apoptotic protein expression. (2) They suppress pathways mediated by cell death receptors. (3) They restrain apoptosis and autophagy by activating the Akt/mechanistic target of rapamycin (mTOR) pathway [4]. The B-cell lymphoma-2 (Bcl-2) family, including Bcl-2 and Bcl-2-associated X protein (Bax), plays an important role in the apoptosis of neuronal cells. Overexpression of Bcl-2 can suppress neuronal apoptosis, whereas overexpression of Bax can promote neuronal apoptosis [80]. Caspase-3 is an essential executor of apoptosis and is regarded as a death protease. By cleaving enzymatically inactivating apoptosis inhibitors and DNA repair-related molecules, Caspase-3 can induce cell death [81]. Neuronal cytochrome C mainly exists in the mitochondria, and an increase in cytochrome C release results in neurosynaptic loss and apoptosis [82].

The anti-apoptotic effects of mycelial polysaccharides from *Armillaria mellea* (AMPS) were studied both in vivo and in vitro. The results indicated that AMPS inhibited intracellular ROS accumulation and nuclear apoptosis, suppressed the activation of caspase-3, and restored mitochondrial membrane potential (MMP) in an HT22 cell apoptosis model induced by L-Glu. AMPS also decreases the apoptosis rate in the hippocampus of AD mice [83]. TL04 is a polysaccharide isolated from *Tremella fuciformis*, with a molecular weight of 2033 kDa, and is mainly composed of Man, Glc, and Rha (5.04:1.87:1). In differentiated PC12 cells induced by glutamate, TL04 was confirmed to inhibit cytochrome c release and Bax expression, increase Bcl-2 levels, and increase the activities of caspase-3, caspase-8 and caspase-9, conferring neuroprotective effects via the caspase-dependent mitochondrial pathway [84]. *Amauroderma rugosum* extract, mainly consisting of polysaccharides, phenolic compounds, and triterpenes, exhibited neuroprotective effects through downregulation of pro-apoptotic proteins and upregulation of Akt/mTOR and mitogen-activated protein (MEK)/ERK pathways, which provides useful information for the prevention and treatment of NDs such as PD [85].

### 4.6. Anti-Neurotoxic Activities of Mushroom Polysaccharides

Glutamate, an acidic amino acid, is the primary excitatory neurotransmitter in the mammalian CNS. Excessive activation of glutamate receptors results in excitotoxicity, and the principal manifestation is the overproduction of ROS, continuous influx of calcium ions, release of pro-apoptotic factors, and mitochondrial dysfunction, which finally cause neuronal dysfunction [86]. The inhibitory effect of mushroom polysaccharides on glutamate-induced cytotoxicity has been reported in some recent studies.

In glutamate-treated PC12 cells, HEP, a polysaccharide obtained from *Hericium erinaceus*, increased cell survival, induced cell differentiation, blocked intracellular Ca^2+^ overload, inhibited ROS production, and prevented MMP depolarization. Moreover, HEP also improved memory impairment and behavioral abnormalities in an AD mouse model induced by D-Gal and AlCl_3_ [87]. However, the mechanisms underlying the neurotoxicity of mushroom polysaccharides require further elucidation.

### 4.7. Anti-Ferroptosis Activities of Mushroom Polysaccharides

Cell death induced by ferroptosis is regarded as a major process leading to neurodegeneration [49]. Therefore, natural compounds with inhibitory effects on ferroptosis as therapeutic candidates for ND treatment have attracted increasing attention from scientists [35]. However, among the various classes of natural products that have been confirmed as ferroptosis inhibitors in NDs, there are few reports on mushroom polysaccharides.

AUM-1 is a polysaccharide isolated from marine *Aureobasidium melanogenum* SCAU-266 with a molecular weight of 8 kDa and Glc, Man, and Gal in a molar ratio of 97.30:1.90:0.08. AUM-1 regulated ferroptosis-related immunomodulatory properties via regulating expressions of proteins related to ferroptosis (GPX4, COX2, FTH1, SLC7A11, and ACLS4) in RAW 264.7 cells [88]. Atractylodes macrocephala Koidz polysaccharides reduced ferroptosis through improving the expression of ferroptosis pathway genes including GPX4, FPN1, FTH1, COX-2, HSPB1, TFR1, NOX1, and ACSL4 in goslings [89]. According to the above analysis, the study of mushroom polysaccharides with inhibitory effects on ferroptosis is promising for the treatment of NDs.

**Table 1:** Effects of mushroom polysaccharides on NDs.

Mechanism	Source (Latin Name)	Polysaccharide	Molecular Weight (kDa)	Monosaccharide Composition	Cell Lines/Model	Type of NDs	Potential Mechanism	Year/References
Anti-oxidant activities	*Inonotus obliquus*	IOPS	111.9	/	L-Glu damaged HT22 cells; APP/PS1 mice	AD	Regulating Nrf2 signaling and exerting antioxidative and antiapoptotic effects	2019 [61]
		IOP	/	/	Tacrine-induced HepG2 cells	AD	Inhibition of ROS generation, 8-OHdG formation in mitochondrial DNA, and loss of the mitochondrial transmembrane potential, decrease in the cytochrome c release and activation of caspase-3	2019 [90]
	*Ganoderma lucidum*	GLP	15.0	/	APP/PS1 mice	AD	Reduce Aβ deposits, increase protein levels of p-FGFR1, p-ERK and p-Akt, potentiate FGFR pathways	2017 [63]
		GLA	/	Gal and Glc (1.0:8.3)	Aβ_25-35_- or Aβ_42_-exposed rat primary cortical neurons	AD	Antagonize Aβ peptide neurotoxicity, inhibit JNK, ERK and p38 MAPK pathways	2008 [64]
		GLP	/	/	Kainic acid-treated Wistar rats	Epilepsy	Inhibit calcium overloading and ERK1/2 and NF-κB expression; stimulate CaMK II α and Cav-1 expression	2015 [65]
	*Ganoderma atrum*	PSG-1	1013	Man, Gal and Glc (1:1.28:4.91)	D-Gal-treated Kunming mice	Aging	Decrease MDA and GSSG levels, increase SOD, CAT, GPx and GSH activities in liver, brain and spleen	2012 [56]
	*Dictyophora indusiata*	DiPS	/	Man, Fuc, Glc, Gal, Rha, Glc-UA and Xyl (86.8:4.5:3.9:1.6:1.2:1.1:0.9)	Caenorhabditis elegans	NDs	Decrease ROS and MDA levels, increase SOD activity, restore MMP and ATP content, regulate DAF-16/FOXO pathways	2016 [26]
	*Pleurotus ostreatus*	POP	/	/	D-Gal and AlCl_3_-treated Wistar rats	AD	Decrease MDA content, increase SOD, GPx and CAT activities in hippocampus, liver and serum	2016 [57]
	*Tricholoma lobayense*	TLH-3	4.23	Rha, Man, Glc-UA, Gal-UA, Glc, Gal and Ara (0.07:0.23:0.02:0.02:1.57:1:0.11)	t-BHP-exposed HELF cells; D-Gal-treated Kunming mice	Aging	Decrease ROS level and inhibit oxidative damage induced by tert-butylhydroperoxide in HELF cells; decrease MDA content and increase SOD and CAT activities in mouse liver and serum	2016 [55]
	*Auricularia auricula-judae*	APP1-a	206	Rha, Ara, Xyl, Man, Glc and Gal (0.2:2.6:0.4:3.6:1.0:0.4)	D-Gal-treated Kunming mice	Aging	Decrease MDA content, increase SOD and GPx activities in liver, serum and heart	2011 [58]
	*Amanita caesarea*	ACPS	18.62	Xyl, Man, Gal and Glc	L-Glu exposed HT22 cells; D-Gal and AlCl_3_-treated balb/c mice	AD	Modulate Nrf2 pathways	2019 [60]
	*Flammulina velutipes*	FVP	/	Man, Rib, Glc, Gal and Xyl (4.07:4.54:3.07:1:2.21)	D-Gal induced Wistar rats	AD	Increase SOD, CAT and GPx levels, decrease MDA levels; anti-apoptosis	2018 [59]
	*Cantharellus cibarius*	CC2a CC3	/	CC2a (Fuc, GlcN, Gal, Glc, Man) CC3 (Glc, Man, Rib)	Human undifferentiated neuroblastoma cell line SH-SY5Y	NDs	Shown antioxidant capacity, effectively neutralize the negative changes induced by activators of glutamatergic system (glutamate, NMDA, AMPA)	2018 [32]
Anti-amyloidogenic effects	*Pleurotus ostreatus*	POP	/	/	D-Gal and Al-treated Wistar rats	AD	Decrease Aβ peptide formation and tau phosphorylation by elevating the expression of PP2A and by reducing the expression of APP, BACE1 and GSK3β	2016 [57]
Anti-neuroinflammation	*Ganoderma lucidum*	GLP	15	/	BV2 microglia and primary mouse microglia; zebrafish	AD	Decrease pro-inflammatory cytokines and promotes anti-inflammatory cytokine expressions in BV-2 and primary microglia; attenuate microglial migration, morphological alterations and phagocytosis probabilities	2017 [71]
	*Antrodia camphorata*	APC	/	/	6-hydroxydopamine treated C57BL/6J mice	PD	Reduce the activation of NLRP3 and the expression of related inflammatory factors	2019 [72]
		APC	/	/	Dopaminergic neuron cell line MES23.5; 6-hydroxydopamine treated mice	PD	Inhibit ROS-NLRP3 signaling	2020 [73]
	*Amanita caesarea*	ACPS2	16.6	Gal, Glc and Man (35.40: 31.77: 29.47)	APP/PS1 mice	AD	Reduce inflammatory cell infiltration in brains, decrease serum concentrations of TNF-α and IL-1β, regulate neuroinflammation by regulating Nrf2 signaling and inhibiting NF-κB activation	2021 [91]
Anticholinesterase activities	*Hericium erinaceus*	HE	/	/	L-Glu-exposed PC12 cells; AlCl_3_ and D-Gal-treated balb/c mice	AD	Enhance the Ach and ChAT concentrations in mouse serum and hypothalamus	2016 [87]
	*Flammulina velutipes*	FVP	/	/	Scopolamine-treated Wistar rats	Cognitive impairment	Elevate the expression of CaMK II and connexin 36, and then regulated the activities of ChAT and AChE to normalize the level of ACh	2015 [92]
	*Armillaria mellea*	AMPS	/	/	L-Glu induced HT22 cell; AlCl_3_ and D-Gal-treated balb/c mice	AD	Increase Ach and ChAT concentrations, decrease AchE concentrations in serum and hypothalamus of mice	2017 [83]
	*Amanita caesarea*	ACPS	18.62	Xyl, Man, Gal and Glc	L-Glu exposed HT22 cells; D-Gal and AlCl_3_-treated balb/c mice	AD	Decrease AchE levels, increase Ach and chAT levels, improve cholinergic neurotransmission	2019 [60]
Anti-apoptotic activities	*Pleurotus eryngii*	PEP	/	/	PC12 Cells induced by β-Amyloid; aging rats	Aging	Decrease intracellular calcium levels, and attenuated the β-amyloid-mediated cell apoptosis in PC12 cells; decrease iNOS, and COX-2 levels in aging rats	2020 [93]
	*Armillaria mellea*	AMPS	/	/	L-Glu induced HT22 cell; AlCl_3_ and D-Gal-treated balb/c mice	AD	enhanced cell viability, suppressed nuclear apoptosis, inhibited intracellular ROS accumulation, prevented caspase-3 activation, and restored MMP	2017 [83]
	*Tremella fuciformis*	TL04	2033	Rha, Man and Glc (1:5.04:1.87)	Glu-induced differentiated PC12 cells	NDs	Enhance Bcl-2 levels, suppress Bax expression and cytochrome c release, decrease activities of caspase-3 caspase-8, caspase-9	2016 [84]
	*Amauroderma rugosum*	AR	/	/	PC12 rat pheochromocytoma cells induced by 6-OHDA	Neurotoxicity	Upregulate the expressions of proapoptotic proteins and downregulate the Akt/mTOR and MEK/ERK dependent pathways	2021 [85]
	*Morchella importuna*	MIP			H_2_O_2_-induced PC12 cells	NDs	Inhibit cell apoptosis via down-regulation of the NF-κB pathway and the p38-JNK pathway and activating of the ERK	2016 [94]
Anti-neurotoxicity activities	*Hericium erinaceus*	HE	/	/	L-Glu induced PC12 Cells; AlCl_3_ and D-Gal-treated balb/c mice	AD	Increase cell survival, induce cell differentiation, block intracellular Ca^2+^ overload, inhibit ROS production and prevent MMP depolarization in L-Glu induced HT22 cell apoptosis model; reduce the apoptosis rate, Aβ deposition, oxidative damage, and p-Tau aggregations in the hippocampus of AD mouse	2016 [87]

## 5. Structure–Activity Relationship of Mushroom Polysaccharides

Polysaccharides isolated from mushrooms are known as glucans. Glucans are made up of D-Glc monomers, and two Glc units can be combined via an α- or β-glycosidic bond. The β (1→3), β (1→6) or α (1→3) linkages form heteroglycans such as Gal, Fuc, Man, Xyl, and arabinose (Ara). They can also form polysaccharide-protein complexes by combining with protein residues [95]. Polysaccharides are not encoded in the genome and their complex structures are closely related to their biological functions. The biological activities of mushroom polysaccharides are influenced by various factors, such as the extraction process of polysaccharides, chemical composition, molecular weight, structural properties, type of molecular linkages, and contents of non-sugar aglycone moieties [35]. Elucidating the structure–activity relationship of mushroom polysaccharides can help us better understand their metabolism in the body.

The antioxidant activities of polysaccharide-protein complexes from *Lentinus edodes* and *G. frondosa* isolated by ultrasound-assisted extraction were generally higher than those obtained by hot water extraction [96]. Three polysaccharides with mean molecular weights of 25.5, 306.2, and 605.4 kDa were obtained from *L. edodes* and named LT1, LT2, and LT3, respectively. In D-Gal-induced aging mice, all three polysaccharides exhibited antioxidant effects by increasing antioxidant enzyme activity and reducing MDA levels. Moreover, LT2 displayed the most effective antioxidant activity among the three aforementioned polysaccharides, indicating a correlation between the molecular weight of the polysaccharides and their antioxidant activities [97]. The antioxidant activities of mycelia zinc polysaccharides (MZPSs) and their main fractions (MZPS-1, MZPS-2, and MZPS-3) isolated from *Pleurotus djamor* have been reported, and MZPS-3 exhibited the strongest antioxidant activity. Further analysis of physicochemical properties revealed that only MZPS-3 contained Xyl and S=O, indicating that the presence of Xyl and S=O could enhance oxidation resistance [98]. Rha and its β configuration may play key roles in maintaining the antioxidant activity of *Lentinula edodes* [99]. Polysaccharides with polyuronic acid residues, β-pyranose, triple helices, and branched structures have also been reported to confer greater antioxidant activities [100]. Structurally modified polysaccharide molecules can also play a more important role than unmodified polysaccharide molecules. Polysaccharide modification methods such as acetylation and sulfation can change the extension of polysaccharides, expose -OH groups, increase the solubility of polysaccharides, and improve the interaction between polysaccharides and specific receptors, thereby enhancing the biological activities of polysaccharides [101]. STLH-3, a sulfated modified polysaccharide from *Phallus chinensis*, showed better antioxidant activity in vitro than the unmodified polysaccharide, TLH-3 [102].

Polysaccharides are an important class of biological polymers with different properties, owing to their various functional groups and distinct monosaccharide compositions. The complex structure of polysaccharides is closely related to their function in living organisms.

## 6. Limitations and Future Perspective

In this review, we discuss the enormous potential of mushroom polysaccharides in the treatment of NDs. However, there are still unresolved issues and limitations in the clinical translation of mushroom polysaccharides into ND therapies. (1) The relationship between neuroprotective activities of mushroom polysaccharides and their structures requires further exploration. Owing to the complicated structures of mushroom polysaccharides, the chemical structures of bioactive polysaccharides are closely related to their biological properties, and polysaccharides purified from the same mushroom exhibit significantly different activities. (2) The lower extraction yield, complex structure, and difficult artificial synthesis of mushroom polysaccharides limit further validation of the mechanism of action. More information on the characteristics of mushroom polysaccharides needs to be obtained with the development of isolation and identification techniques [81]. (3) Although previous studies have confirmed the neuroprotective activities of these bioactive molecules in multiple animal models, further elucidation is still needed regarding in vivo pharmacokinetics such as the metabolism and absorption of mushroom polysaccharides. New technologies, including metabolomics and transcriptomics, are required to provide new methodological tools and clarify the underlying mechanisms. (4) The degradation occurring at the level of first-pass metabolism and absorption from the gastrointestinal tract may lead to low bioavailability of mushroom polysaccharides, which needs to be addressed in further studies. (5) At present, the reported neuroprotective effects of mushroom polysaccharides are scarce, and new bioactive polysaccharides from various mushrooms need to be identified. (6) Neuroprotective effects of mushroom polysaccharides have mainly been observed in cell and animal models, which are hardly related to the existence of mushroom polysaccharides in the human body. To provide more direct clinical evidence and support their therapeutic benefits in patients with NDs, large randomized clinical studies on mushroom polysaccharides are needed to provide a more direct clinical evidence and support their therapeutic benefits in patients with NDs. In addition, in clinical trials of mushroom polysaccharides as drugs for NDs, we need to prioritize their safety and efficacy.

## 7. Conclusions

This review summarizes the current knowledge around mushroom polysaccharides with neuroprotective activities and emphasizes their main roles and molecular mechanisms in treating NDs. The present findings may provide options for mushroom polysaccharides as promising neuroprotective agents.

## Data Availability

Not applicable.

## Supplementary Materials

The following supporting information can be downloaded at: https://www.mdpi.com/article/10.3390/nu14224833/s1, Table S1: Effects and side effects of medications for the treatment of NDs [103,104].

## Abbreviations

6-hydroxydopamine (6-OHDA); Acetylcholine (ACh); Acetylcholinesterase (AChE); Adenosine triphosphate (ATP); Alzheimer’s disease (AD); Amyloid beta (Aβ); Amyloid precursor protein (APP); Amyloid precursor protein/presenilin 1 (APP/PS1); Amyotrophic lateral sclerosis (ALS); Arabinose (Ara); B-cell lymphoma-2 (Bcl-2); Bcl-2-associated x (Bax); Beta-secretase 1 (BACE-1); Butyrylcholinesterase (BChE); Catalase (CAT); Central nervous system (CNS); Choline acetyltransferase (ChAT); c-Jun N-terminal kinase (JNK); Decay accelerating factor-16 (DAF-16); Extracellular regulated protein kinase (ERK); Forkhead box O (FOXO); Frontotemporal dementia (FTD); Fucose (Fuc); Galactose (Gal); Galacturonic acid (Gal-UA); Glucose (Glc); Glucuronic acid (Glc-UA); Glutamine (Glu); Glutathione (GSH); Glutathione peroxidase 4 (GPx4); Huntington’s disease (HD); Kilodalton (kDa); Lewy body dementia (LBD); Malondialdehyde (MDA); Mannose (Man); Mechanistic target of rapamycin (MTOR); Mitochondrial membrane potential (MMP); Mitogen activated protein (MEK); Mitogen activated protein kinase (MAPK); Neurodegenerative diseases (NDs); Nitric oxide (NO); Nuclear factor erythroid 2-related factor 2 (Nrf2); Nuclear factor kappa-B (NF-κB); Nucleotide-binding domain, leucine-rich-containing family, pyrin domain containing 3 (NLRP3); Oxidative stress (OS); Parkinson’s disease (PD); Protein kinase B (Akt); Reactive oxygen species (ROS); Rhamnose (Rha); Ribose (Rib); Superoxide dismutase (SOD); Xylose (Xyl).
