# Supplementary material for: Mushroom Polysaccharides as Potential Candidates for Alleviating Neurodegenerative Diseases
Source: Nutrients. 2022 Nov 15;14(22):4833. doi: 10.3390/nu14224833 (PMC9696021; doi:10.3390/nu14224833)
Supplement: Supplementary file 1 [file nutrients-14-04833-s001.zip › nutrients-2026548-supplementary.pdf]

molecules MDPI

Table S1. Effects and side effects of medications for the treatment of NDs.

NDs	Types	Drug	Effects	Adverse effect	Reference
AD	Cholinesterase inhibitors	Galantamine, memantine, rivastigmine, donepezil and tacrine	Inhibiting the activity of AChE, thereby increasing the cholinergic levels in the brain	Convulsions, vomiting, nausea, diarrhea, stomach cramps, liver damage, weight loss, chest pain, muscle cramps and weakness, irregular breathing and heartbeat	[1]
	Anti-oxidant drugs	Melatonin, monoamine oxidase inhibitors	Anti-oxidation, anti-inflammatory effects, inhibitory effects on the Aβ generation	Hypertensive crisis, hypertension	[2,3]
	Calcium channel blockers	Flunarizine, nimodipine, nilvadipine, nitrendipine	Decreasing calcium influx through the plasma membrane and impairment of synapse physiology, protecting AD cells from Aβ oligomer production	Affecting normal neurotransmission	[4]
	Drugs directed at β1 amyloid	MK-8931, E2069	Decreasing the concentrations of cerebral Aβ	Seizures, hypomyelination, axon guidance defects, neurogenesis abnormalities, memory deficits	[5]
PD	Catechol-O-methyltransferase inhibitors	Entacapone, tolcapone, opicapone	Inhibiting catechol-O-methyl transferase enzyme, leading to a higher levodopa concentration in the blood	Liver toxicity, drowsiness, nausea, insomnia, hallucination, dizziness	[6]
	Drugs for stimulating dopaminergic signaling	Levodopa, DOPA decarboxylase inhibitors	Being converted to dopamine and stored in the vesicles of presynaptic dopaminergic neurons	Abnormal involuntary movements and results in levodopa-induced dyskinesia	[7]
	Inhibitors of the monoamine oxidase B	Rasagiline, selegiline	Degrading the neurotransmitter dopamine that is deficient in the nigro-striatal region in PD, and forms H_2O_2 and toxic aldehyde metabolites of dopamine	Vomiting, nausea, hypotension	[8]

	Dopamine agonists	Apomorphine, ergot alkaloids, ropinirole	Induce an antiparkinsonian effect through actions on either D1-like or D2-like dopamine receptors	Producing dyskinesia identical to that of levodopa	[9,10]
HD	Inhibitors of the vesicular monoamine transporter type 2	Deutetrabenazine, tetrabenazine	Inhibiting vesicular monoamine transporter type 2, decreasing available dopamine in the synapse and interacting with postsynaptic dopamine receptors	Parkinsonism, depression, akathisia, anxiety, fatigue	[11]
ALS	Anti-excitotoxic drug	Riluzole	Blocks the presynaptic release of glutamate	Nausea, asthenia, vomiting, diarrhoea, anorexia, dizziness, increased alanine transferase, low hemoglobin	[12]
	Anti-oxidant drug	Edaravone	A free radical scavenger to reduce oxidative stress	Abnormal liver function, increased alanine transferase	[13]

References

1. Sharma, K. Cholinesterase inhibitors as Alzheimer's therapeutics. *Molecular Medicine Reports* **2019**, *20*, 1479-1487, doi:10.3892/mmr.2019.10374.
2. Ostadkarampour, M.; Putnins, E.E. Monoamine Oxidase Inhibitors: A Review of Their Anti-Inflammatory Therapeutic Potential and Mechanisms of Action. *Frontiers in Pharmacology* **2021**, *12*, doi:10.3389/fphar.2021.676239.
3. Cardinali, D.P.; Vigo, D.E.; Olivar, N.; Vidal, M.F.; Brusco, L.I. Melatonin Therapy in Patients with Alzheimer's Disease. *Antioxidants (Basel)* **2014**, *3*, 245-277, doi:10.3390/antiox3020245.
4. Nimmrich, V.; Eckert, A. Calcium channel blockers and dementia. *British Journal of Pharmacology* **2013**, *169*, 1203-1210, doi:10.1111/bph.12240.
5. Blume, T.; Filser, S.; Jaworska, A.; Blain, J.F.; Koenig, G.; Moschke, K.; Lichtenthaler, S.F.; Herms, J. BACE1 Inhibitor MK-8931 Alters Formation but Not Stability of Dendritic Spines. *Front Aging Neurosci* **2018**, *10*, 229, doi:10.3389/fnagi.2018.00229.
6. Salamon, A.; Zádori, D.; Szpisjak, L.; Klivényi, P.; Vécsei, L. What is the impact of catechol-O-methyltransferase (COMT) on Parkinson's disease treatment? *Expert opinion on pharmacotherapy* **2022**, *23*, 1123-1128, doi:10.1080/14656566.2022.2060738.
7. Bandopadhyay, R.; Mishra, N.; Rana, R.; Kaur, G.; Ghoneim, M.M.; Alshehri, S.; Mustafa, G.; Ahmad, J.; Alhakamy, N.A.; Mishra, A. Molecular Mechanisms and Therapeutic Strategies for Levodopa-Induced Dyskinesia in Parkinson's Disease: A Perspective Through Preclinical and Clinical Evidence. *Front Pharmacol* **2022**, *13*, 805388, doi:10.3389/fphar.2022.805388.
8. Nagatsu, T.; Sawada, M. Molecular mechanism of the relation of monoamine oxidase B and its inhibitors to Parkinson's disease: possible implications of glial cells. *Journal of neural transmission. Supplementum* **2006**, 10.1007/978-3-211-33328-0_7, 53-65, doi:10.1007/978-3-211-33328-0_7.
9. Jenner, P. Pharmacology of dopamine agonists in the treatment of Parkinson's disease. *Neurology* **2002**, *58*, S1-8, doi:10.1212/wnl.58.suppl_1.s1.
10. Sit, S.Y. Dopamine agonists in the treatment of Parkinson s disease past, present and future. *Current pharmaceutical design* **2000**, *6*, 1211-1248, doi:10.2174/1381612003399581.
11. Potkin, K.T.; Potkin, S.G. New directions in therapeutics for Huntington disease. *Future neurology* **2018**, *13*, 101-121, doi:10.2217/fnl-2017-0035.
12. Miller, R.G.; Mitchell, J.D.; Lyon, M.; Moore, D.H. Riluzole for amyotrophic lateral sclerosis (ALS)/motor neuron disease (MND). *Amyotrophic Lateral Sclerosis* **2003**, *4*, 191-206.
13. Chio, A.; Mazzini, L.; Mora, G. Disease-modifying therapies in amyotrophic lateral sclerosis. *Neuropharmacology* **2020**, *167*, 107986, doi:10.1016/j.neuropharm.2020.107986.
